# Overview of Control Programs for Cattle Diseases in Finland

**DOI:** 10.3389/fvets.2021.688936

**Published:** 2021-07-30

**Authors:** Tiina Autio, Erja Tuunainen, Hannele Nauholz, Hertta Pirkkalainen, Laura London, Sinikka Pelkonen

**Affiliations:** ^1^Finnish Food Authority, Veterinary Bacteriology and Pathology Unit, Kuopio, Finland; ^2^Animal Health ETT, Seinäjoki, Finland; ^3^Finnish Food Authority, Virology Unit, Helsinki, Finland

**Keywords:** cattle diseases, control program, SOUND control, Finland, bovine

## Abstract

Animal disease control has a long tradition in Finland. The country is free of all EU-regulated cattle diseases of categories A and B. Infectious bovine rhinotracheitis, enzootic bovine leucosis, bovine viral diarrhea, bluetongue, bovine genital campylobacteriosis, and trichomoniasis do not currently exist in the country. The prevalence of paratuberculosis, *Mycoplasma bovis*, salmonella infection, and Q-fever is low. The geographic location, cold climate, low cattle density, and limited animal imports have contributed to the favorable disease situation. Besides screening for selected regulated diseases, the national disease-monitoring program includes periodic active monitoring of non-regulated diseases, which allows assessment of the need for new control measures. The detection of diseases through efficient passive surveillance also plays an important part in disease monitoring. The Finnish cattle population totals 850,000 animals kept on 9,300 cattle farms, with 62,000 suckler cows in 2,100 herds and 260,000 dairy cows in 6,300 herds. Animal Health ETT, an association owned by the dairy and meat industry, keeps a centralized cattle health care register. Animal Health ETT supervises cattle imports and trade within the country and runs voluntary control programs (CP) for selected diseases. Active cooperation between authorities, the cattle industry, Animal Health ETT, and herd health experts enables the efficient planning and implementation of CPs. CPs have been implemented for cattle diseases such as salmonella, *Mycoplasma bovis*, ringworm, and *Streptococcus agalactiae*. The CP for salmonellosis is compulsory and includes all Salmonella serotypes and all cattle types. It has achieved the goal of keeping the salmonella prevalence under 1% of cattle herds. CPs for *M. bovis*, ringworm, and *S. agalactiae* are on a voluntary basis and privately funded. The CP for *Mycoplasma* was designed in collaboration with national experts and has been implemented since 2013. The CP includes observation of clinical signs, nasal swab sampling from calves, and bulk tank milk and clinical mastitis samples for *M. bovis*. *M. bovis*-negative herds gradually achieve lower status levels for *M. bovis* infection. The general challenge facing voluntary CPs is getting farms to join the programs.

## Introduction

**Figure 1:**
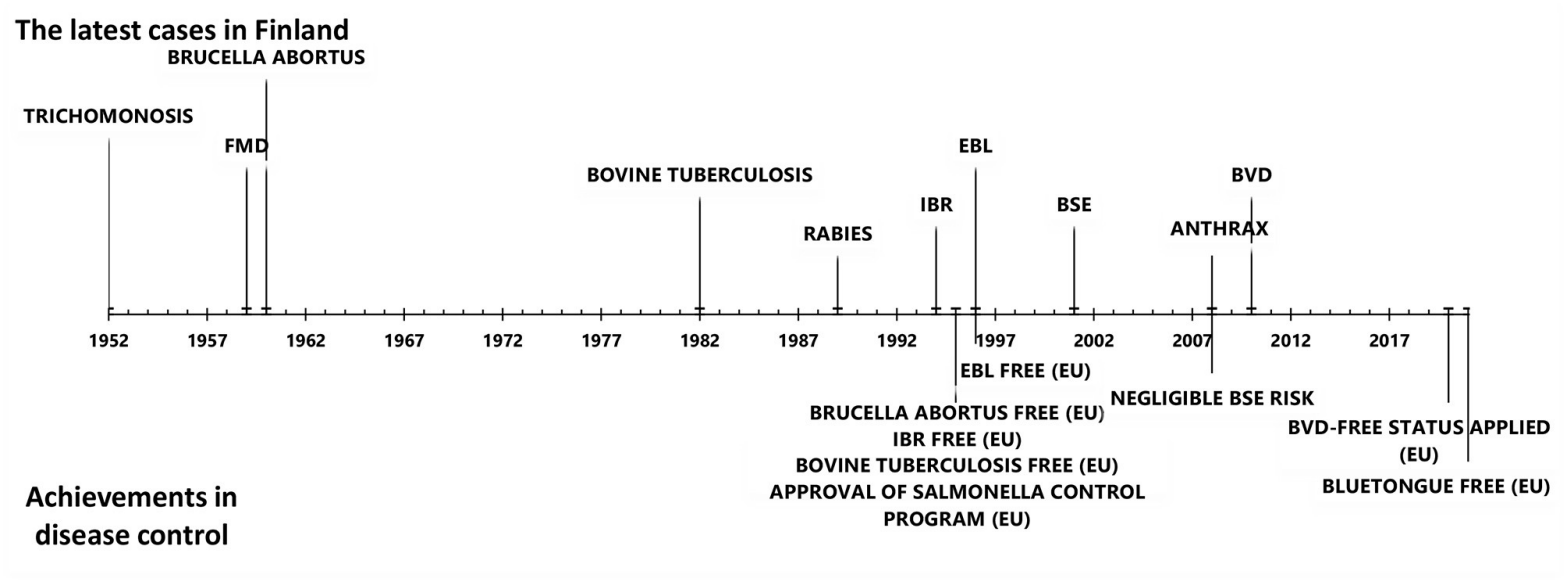
The latest cases of important cattle diseases and achievements in cattle disease control in Finland since 1950.

Animal disease control has a long tradition in Finland. The geographical location, cold climate, low cattle density, restricted animal imports before joining the EU in 1995, and strict control of imports thereafter have contributed to the favorable disease situation. Finland is free of all EU-regulated cattle diseases (diseases in categories A and B), including foot and mouth disease, rinderpest, rift valley fever, bovine brucellosis, bovine tuberculosis, rabies, lumpy skin disease, and contagious bovine pleuropneumonia. In addition, infectious bovine rhinotracheitis (IBR), enzootic bovine leucosis (EBL), bovine viral diarrhea (BVD), bluetongue (BT), bovine genital campylobacteriosis, and trichomoniasis do not currently exist in Finland (Figure 1). Furthermore, the prevalence of *Mycoplasma bovis*, salmonella infection, paratuberculosis, and Q-fever is low.

Nationwide screening of diseases from bulk tank milk (BTM) samples and blood samples from suckler cow herds in slaughterhouses has been used in disease monitoring. Subsequently launched control programs, either voluntary or compulsory, have led to a decreased prevalence of disease and, finally, to disease eradication. Finland has succeeded in eradicating IBR, EBL, and BVD (Figure 1). Moreover, a mandatory control program for salmonella, in act since 1995, has documented an exceptionally low prevalence of salmonella.

In this paper, we describe the current Finnish control measures and control programs for cattle diseases for which control programs have been implemented in two or more regions in the EU (1). The diseases were selected in the framework of the SOUND control project (COST Action Standardizing Output-based Surveillance to Control Non-Regulated Diseases in the EU, https://sound-control.eu). We also present the characteristics of disease surveillance and cattle production in Finland, which have enabled the good cattle disease situation.

## Materials and Methods

Cattle production data were obtained from the agricultural statistics of the Natural Resources Institute Finland (2), the official Bovine Register (3), statistics of the International Committee for Animal Recording (ICAR) (4), Eurostat (5), and the Finnish Dairy Herds Recording System (6). Animal health data, diagnostic results for cattle diseases, annual monitoring plans of governmental control programs, and meat inspection records were obtained from the Finnish Food Authority (FFA) (7, 8). Data concerning privately funded control programs were obtained from Animal Health ETT and the Naseva register (9).

## Cattle Production in Finland

The Finnish cattle population is ~850,000, raised on a total of 9,300 farms (2, 3). There are 260,000 dairy cows on 6,300 farms. The average herd size is 50 cows among herds in the Finnish Dairy Herd Recording System (4). The number of dairy farms has decreased during the last 10 years, while the average herd size has increased, and this trend appears to be continuing. A special characteristic is the raising of coeval bull calves for meat production using the all-in-all-out principle. Calves originating from several dairy farms are transported to specialized calf-rearing units, where they are housed in group pens typically containing 10–60 calves. At the age of 6 months, the entire group is moved into finishing units. There are 62,000 suckler cows on Finnish cattle farms, and 1,600 farms have only suckler cows (2, 3). Most cattle farms raise only cattle and are very seldom (2.2%) mixed.

The overall density of cattle is rather low, being 0.5 livestock unit (LSU) per hectare (5). However, there are few farms in the north of Finland, and cattle production is clustered in the central parts of the country. The cattle are mainly fed on farm-grown grass and some amounts of grains and rapeseed/canola.

## Administration of Animal Disease Control in Finland

**Figure 2:**
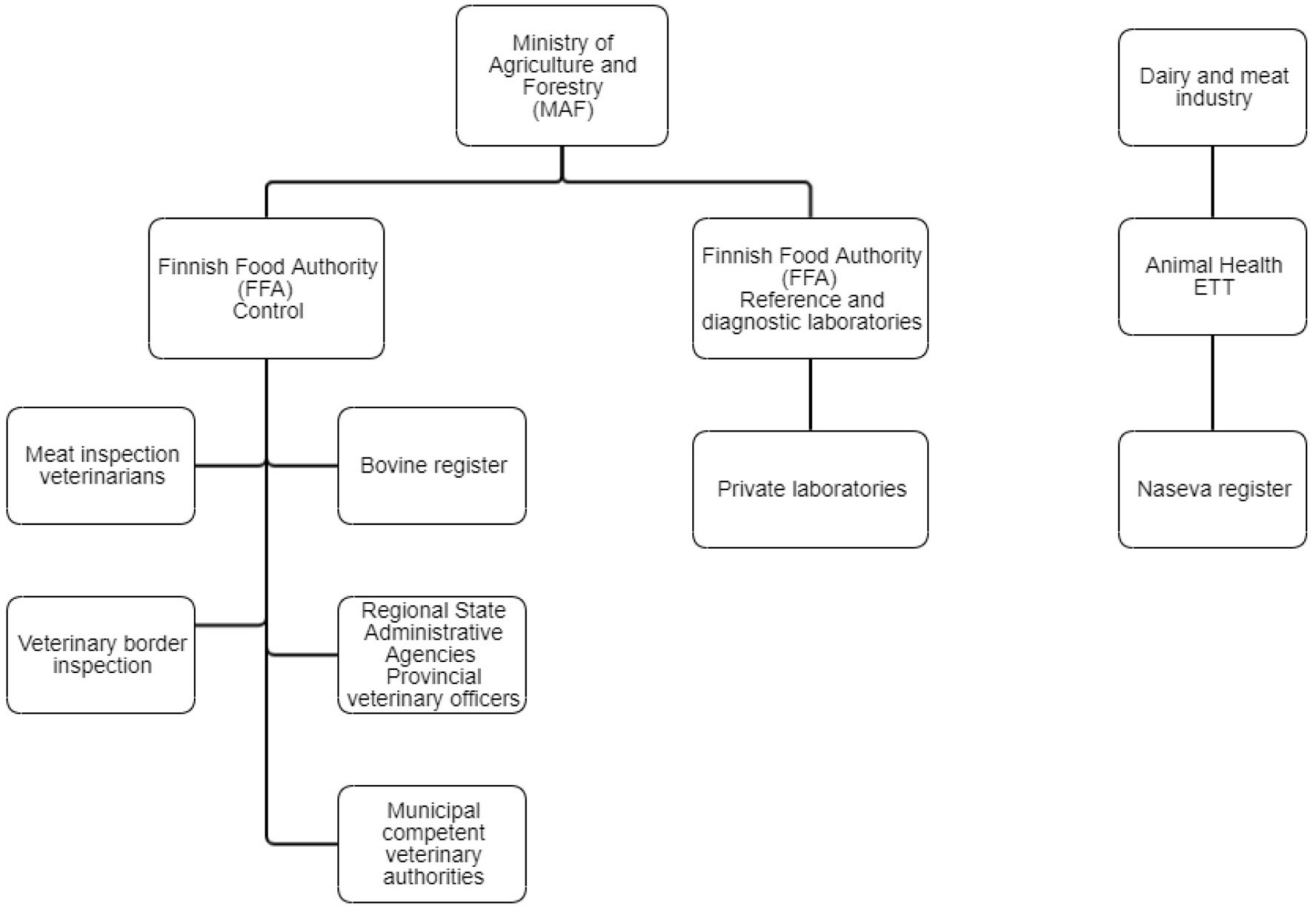
Organizations involved in cattle disease control in Finland. The Naseva register has several interfaces to databases and different computer systems, e.g., the Bovine Register, mastitis testing laboratories, the Finnish Dairy Herd Recording System, meat inspection databases of slaughterhouses, and the veterinary practice management systems.

The highest authority in controlling animal diseases is the Ministry of Agriculture and Forestry (MAF) (Figure 2). National acts and orders on several animal diseases enacted by MAF include, for example, control measures, notification procedures, and prohibition of vaccination. The control division of the FFA directs and monitors the implementation of and compliance with legislation. Veterinary border inspection and meat inspection belong to its control activities, as well as the registration of animals. The FFA steers disease control activities in Regional State Administrative Agencies, which, in turn, direct the function of competent veterinary authorities. The FFA draws up an annual nationwide plan of monitoring programs for animal diseases, and it directs and oversees the implementation of the monitoring programs by issuing orders on sampling to Regional State Administrative Agencies or directly to dairy companies and slaughterhouses. The laboratory and research division of the FFA performs reference laboratory functions as well as disease diagnostics. The FFA publishes an annual report of animal diseases, including monitoring data on infectious diseases (7, 8).

## Categorization of Cattle Diseases in Legislation

Cattle diseases fall into different control categories in Finnish legislation. All cattle diseases listed under category A, B, and C of the European Animal Health Law are controlled by the government, and reimbursements are paid to farmers if animals are culled. Of the diseases listed in categories C–E, the most important in the country are categorized as “to be combated” according to legislation, including IBR, EBL, BVD, BT, anthrax, and salmonella. These are controlled by the government, and for some of them, reimbursements are paid for the culled animals. If a disease to be combated is suspected on a cattle holding, the herd owner must inform a competent veterinary authority in accordance with the Animal Diseases Act 476/2021 (10). If a positive animal is detected, control measures are applied, including the restriction of animal movements and culling of cattle in the herd, depending on the disease.

Some diseases regarded as less serious in the country fall into the category “to be reported” by the veterinarian to the competent authorities. These are voluntarily controlled by the farmer and there is sometimes a voluntary control program or other control measures organized by the industry (such as for *Mycoplasma bovis*, paratuberculosis, and ringworm).

Samples related to combated diseases must be analyzed in the laboratory of the FFA, and salmonella is also analyzed in an official accredited laboratory. Positive samples or microbes isolated from diseases to be reported must be sent to the reference laboratory (FFA) for epidemiological surveillance, if analyzed in other laboratories.

## Monitoring of Cattle Diseases

In the monitoring of various bovine diseases, there is a long tradition of sampling in dairies and slaughterhouses (Tables 1, 2). BTM sampling of dairy herds is performed by dairies, and blood sampling of suckler cow herds in slaughterhouses. The FFA requests sampling of cattle *via* the Bovine Register. Sampling requests are observed by slaughterhouses prior to the slaughter of animals during mandatory register checks. Both random and risk-based samplings are utilized in the selection of herds for surveillance. In the risk-based sampling of diseases causing abortions, such as IBR, BVD, brucella, and Q-fever, herd selection is based on gestation data from the Finnish Dairy Herd Recording System. Herds with elevated numbers (>5%) of abortions or short gestation periods are sampled.

**Table 1:** BTM and serum sampling for surveillance of BVD, IBR, and EBL in Finnish cattle herds.

		**No. of serum samples (no. of holdings, suckler cows) tested**			**No. of BTM samples tested**		
**Year**	**Total no. of holdings (suckler cows)^a^**	**BVD**	**IBR**	**Total no. of holdings (dairy)^b^**	**BVD**	**IBR**	**EBL**
2010	1,511	4,108 (609)	4,108 (609)	11,933	11,112	3,277	3,277
2011	1,504	4,661 (698)	4,661 (698)	11,259	3,302	1,449	1,449
2012	1,520	5,096 (715)	5,096 (715)	10,584	2,963	1,312	1,312
2013	1,513	2,485 (469)	2,485 (469)	9,993	1,800	1,292	1,292
2014	1,495	7,915 (991)	7,915 (991)	9,499	1,277	1,277	1,277
2015	1,499	8,141 (1,006)	8,141 (1,006)	9,039	989	989	989
2016	1,494	7,901 (950)	7,901 (950)	8,519	920	920	920
2017	1,524	6,885 (992)	6,885 (992)	7,921	715	715	715
2018	1,546	1,832 (365)	1,832 (365)	7,374	1,255	1,255	1,255
2019	1,566	1,970 (331)	1,970 (331)	6,755	1,344	1,344	1,214
2020	1,566	2,450 (410)	2,450 (410)	6,314	1,298	1,298	1,298

a *Number of holdings that have suckler cows but no dairy cows*.

b *Number of holdings that have at least one dairy cow*.

**Table 2:** Surveillance of BVD, IBR, and EBL in Finnish cattle herds in 2019.

	**BVD**		**IBR**		**EBL**
**Survey**	**Antibodies**	**PCR**	**Antibodies**	**PCR**	**Antibodies**
Dairy herd BTM sampling	1,344		1,344		1,214
Random sampling of dairy herds	591		591		
Risk-based sampling of dairy herds	753		753		
Random sampling of suckler cow herds	1,970		1,970		
Sampling related to artificial insemination	157	106	157		157
Passive surveillance	126	99	126	98	133
Import (e.g., live animals, semen, embryo recipient cows)	108	45	62	21	3
Other reasons (e.g., trade, export)	85		4		1
Total	3,790	250	3,663	119	1,508

To control mastitis-causing pathogens, monitoring of causative agents is of major importance. Extensive screening for mastitis pathogens in individual milk samples from cases of clinical and subclinical mastitis has been conducted for decades in Finland. In 2020, ~170,000 quarter milk samples (QMS) were tested for mastitis pathogens (there are ~260,000 dairy cows in Finland) (9). Multiplex real-time PCR targeting several mastitis pathogens, including *M. bovis, Staphylococcus aureus*, and *Streptococcus agalactiae*, has been in use since early 2012 (Pathoproof® Complete 16-kit, Thermo Fisher Scientific, Finland).

## Centralized Cattle Health Care Register (Naseva Register)

The Voluntary Centralized Cattle Health Care Register (Naseva register) was developed in 2005 in cooperation with dairy companies and slaughterhouses (9). The Naseva register is administrated by an industry-based association, Animal Health ETT. At the end of 2020, a total of 93% of Finnish dairy farms and 90% of meat production farms were included in the Naseva register. The classification of Finnish dairy and suckler cow herds and requirements for each level are described in Table 3. The Naseva register is used to document, manage, and produce data related to food safety, animal health, and welfare by dairies, slaughterhouses, cattle farms, veterinarians, and other authorized partners. The Naseva register has several interfaces to databases and different computer systems, such as the Bovine Register, mastitis testing laboratories, the Finnish Dairy Herd Recording System, meat inspection databases of slaughterhouses, and veterinary practice management systems (3, 6, 9). Farms can be tagged in the Naseva register if there is a disease outbreak, a suspicion of contagious diseases, positive test results, or another unusual event in the herd. These tags are on display, for example, to slaughterhouses and animal brokers and are used to plan the grouping and transportation of animals to calf-rearing units.

**Table 3:** Classification of Finnish dairy and suckler cow herds in the Naseva register and in the *M. bovis* control program.

	**Classification of herds in Naseva Register**			
**Requirements**			***M. bovis*** **control program**	
	***M. bovis* infected herds during control measures**	**Naseva national level herds**	**Joining level (B level) herds**	**A level herds**
**Herd health**				
Veterinarian herd health visits	Minimum 2/year	Minimum 1/year	Minimum 2/year	Minimum 2/year
Veterinarian monitores the herd health and meat inspection data in Naseva Register	Yes	Yes	Yes	Yes
Health care and Biosecurity plan	Yes	Yes	Yes	Yes
Medication data documented in Naseva Register	Voluntary	Voluntary	Mandatory	Mandatory
Risk assesment or use of Biocheck.UGent®	Yes	Voluntary	Yes	Yes
Presence of *M. bovis* infections	Yes	No	No	No
**Sampling for** ***Mycoplasma bovis***				
Sampling of heatlhy calves for *M.bovis* (PCR or Elisa)^a^	Three sampling occasions with negative results to reach National level^b^	No	Twice^b^	2/year (dairy), 1/year (suckler cows)
Testing of BTM for *M. bovis* by PCR	Yes^c^	No	Twice^b^ (dairy)	2/year (dairy)
Routine testing of QMS for mastitis pathogens (by PCR including *M. bovis*)	Yes	Recommended	Yes	Yes
Sampling of clinical cases	Yes	Yes	Yes	Yes
**Control of cattle movements**				
Movements of cattle from the herd	Only to infected calf rearing or finishing units and slaughter	Yes	Yes	Yes
Use of health certificates in cattle trade	Recommended	Recommended	Mandatory	Mandatory
Purchased cattle tested for salmonella	Mandatory	Mandatory	Mandatory	Mandatory
Regular testing of mastitis QMS (*S. agalactiae, M. bovis*) and BRD (*M. bovis*) cases in herd of origin	Recommended	Recommended	Mandatory	Mandatory
Screening the herd of orgin for symptoms of *M. bovis*, paratuberculosis, contagious hoof diseases, ringworm	Recommended	Recommended	Mandatory	Mandatory
Partipating in cattle shows	Not allowed	Not recommended	Only shows of A level herds	Only shows of A level herds

a *Nasal swab sampling of all (max 20) calves of age 1 week-6 months; in herds with less than 10 calves additional antibody testing of 15 animals over 3 months*.

b *In 4-8 months interval*.

c *During control measures recommended to test 1-2/week until negative results, followed by monthly testing*.

When joining the Naseva register, a farmer makes a contract with the herd health veterinarian in the register. A minimum of one annual herd health visit, including a documented health care plan, is required for each herd. More frequent visits are needed, for example, in control programs and in relation to the delivery of medicine. The content of health care visits has been defined by the national veterinary health care expert group. The visits include the monitoring of production data and animal movements, and observation of the condition, health, and behavior of different age groups, which are conducted in accordance with the Welfare Quality® (11) principles. The mortality in different age groups can be evaluated online. The occurrences of symptoms and cases of salmonellosis, paratuberculosis, *S. agalactiae*, diarrhea, abortions, respiratory diseases, contagious hoof diseases, *M. bovis*, and *Trichophyton verrucosum* are monitored, and sampling is suggested when needed. Disease control measures, feed hygiene, and biosecurity are also evaluated by the veterinarian.

## Import of Bovine Animals

**Figure 3:**
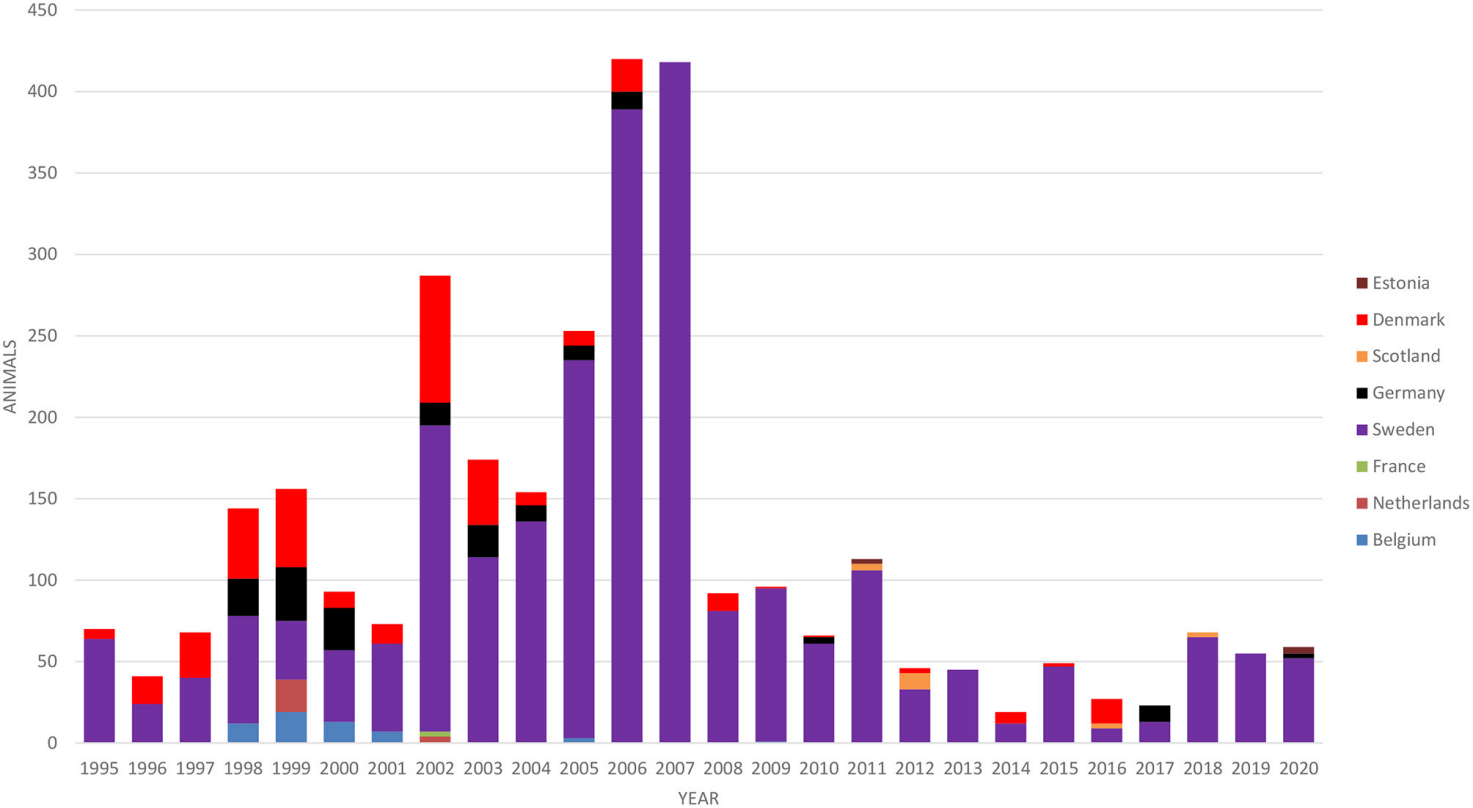
Number of cattle imported to Finland according to the country of origin in 1995–2020.

Cattle imports to Finland are very limited, mainly comprising a small number of breeding animals. The numbers of imported cattle according to the country of origin in 1995–2020 are presented in Figure 3. The majority of animals (79%) were breeding animals for suckler cow herds. Most of the imported cattle originated from Sweden (80% of all imports in 1995–2020), where BVD has been eradicated, paratuberculosis is well-controlled, and the *M. bovis* situation is similar to that in Finland. Dairy cattle were only imported from Sweden in 1995–2020.

All cattle imported to Finland must be tested or come from countries free of bovine tuberculosis, brucellosis, BT, and EBL, and they must fulfill the requirements set in EU regulation (EU 2020/688). This also applies to IBR in Finland (12).

The importers are also instructed by Animal Health ETT. The main principle of guidance is to direct importers to purchase animals from countries with a similar cattle disease situation. All imported cattle should be isolated for 30 days before entering the herd. Animals should be tested for BVD antibodies and BVD virus, both in the country of origin and when arriving in Finland. Cattle traded from Sweden only need to be tested after arrival, but the farm of origin must be free of BVD and belong to the BVD-monitoring program. All animals must be tested for salmonella, and the disease status of *M. bovis, T. verrucosum*, paratuberculosis, and *Leptospira hardjo* in the herd/country of origin is evaluated, and testing is instructed as appropriate. Vaccination against *T. verrucosum* is recommended. The instructions have been voluntarily followed by all importers who have a production contract with dairy and slaughterhouse companies.

## Biosecurity on Farms

The greatest risk of introducing new diseases into a herd is caused by purchased animals. Because of the low cattle density in Finland, other contacts between animals from different farms are almost non-existent, and common pastures are a rarity. Moreover, there is no tradition of public livestock markets and auctions.

Animal trading between Finnish herds belonging to the Naseva register is strictly supervised by Animal Health ETT. Farmers obtain electronic farm health reports and health certificates from the Naseva register to ensure safe animal trade. Approximately 80% of farmers purchasing cattle use health certificates in the purchase. The animals should not be moved until the buyer has accepted the report and an optional veterinary certificate. The latest veterinary health care visit should have been within 3 months of the trade. No animals with a lower or unknown health status can be accepted in the transport.

The health requirements of animal trade (Table 3) include the following: Purchased animals are clinically healthy and have tested negative for salmonella within 2 months. Udder and respiratory tract infections in the herd have been tested with PCR for *S. agalactiae* and *M. bovis* with negative results. No symptoms of contagious hoof diseases or ringworm have been observed in the previous 3 years and no diarrhea or respiratory disease in the previous 1 month in the herd. There have been no signs of *M. bovis* infection or paratuberculosis in veterinary health care visits. Risk management guidelines have been followed if needed in the herd. Preference should be given to herds in the *M. bovis* control program.

Advice on and evaluation of biosecurity on farms are an important part of health visits to herds in the Naseva register. Of these farms, 90% have separate clothing for authorized visitors and a possibility to wash hands before entering the barn. The Biocheck.UGent® (13) evaluation protocol was integrated in the Naseva register in April 2021 and is available to the veterinarian for herd health visits.

Sharing of breeding bulls only occurs in a very small number of herds in semi-intensive cattle production, while most herds use artificial insemination.

Cattle shows are rare, with <10 being organized annually. Instructions regarding the health status of herds of origin and the participating animals are provided by the FFA and Animal Health ETT. The recommendations include the absence of salmonella, *M. bovis, S. agalactiae*, ringworm, and contagious hoof diseases.

## Control of Cattle Diseases in Breeding Bulls

Breeding bulls used for AI semen collection must be obtained from a holding free of *Mycobacterium tuberculosis* complex, brucellosis, EBL, IBR, and salmonella. Before quarantine, animals must be tested for infection with *M. tuberculosis* complex, brucellosis, EBL, IBR, and BVD (14). During quarantine, bulls are tested for brucellosis, IBR, BVD, salmonella, *Campylobacter fetus* spp. *venerealis*, and *Tritrichomonas fetus* (14, 15).

## Control Programs for Cattle Diseases in Finland

The following diseases have control programs in at least two regions within the EU (1). Here, we present the diseases in two sections: (i) diseases never detected in or eradicated from Finland and (ii) diseases present sporadically or endemically in Finland. These diseases belong to different control categories in Finnish legislation, for example, diseases to be combated and to be reported (16).

### Control of Cattle Diseases Never Detected in or Eradicated From Finland

#### Infectious Bovine Rhinotracheitis

Finland is officially free of IBR and has had additional guarantees in cattle trade in the EU since 1994 (12). The first BHV-1 infection was recorded in 1970 and was presumably imported in 1968. Large-scale BTM surveillance started in 1990, and the disease was eradicated in 1994 (17, 18). The eradication of IBR from Finland has been described in detail by Nuotio et al. (17).

The governmental compulsory control program to prove an official disease-free status is based on nationwide annual antibody surveys both from BTM and serum samples (Table 1). The BTM survey consists of both random and risk-based sampling of dairy herds with elevated levels of abortions. Suckler cow herds are randomly blood sampled in slaughterhouses. In addition, animals intended for artificial insemination are tested (14). All aborted fetuses sent to a diagnostic laboratory are tested for IBR by PCR, and serum samples from aborted cows are examined for IBR antibodies. The numbers of tested samples and cattle herds in 2019 are presented in Table 2.

IBR is a disease to be combated, and suspicions and detected cases are dealt with by regional and local official veterinarians. Vaccinations are prohibited (19).

#### Enzootic Bovine Leukosis

Finland is officially free of EBL (12, 19). The Finnish mainland was given an official EBL-free status in 1996 according to Council Directive 64/432/EEU, and the island district of Ahvenanmaa followed in 1999. A single antibody-positive animal was detected in 2008 (20). Eradication of EBL from Finland has been described in detail by Nuotio et al. (21). In brief, the key principle was test and slaughter.

The governmental compulsory control program is based on a nationwide annual BTM antibody survey. Since 2011, the BTM survey of dairy farms has been based on random sampling (8). In addition, samples are tested from animals intended for artificial insemination (14). The numbers of tested samples and cattle herds are presented in Tables 1, 2. In addition, lesions in which EBL is suspected on meat inspection must be tested by histopathological examination and animals using serological tests (19).

EBL is a disease to be combated, and suspicions and detected cases are dealt with by regional and local official veterinarians.

#### Bovine Viral Diarrhea

Finland has been free of BVD since 2010, and an application for an official disease-free status is under evaluation in the EC. The last case was detected in 2010 (22), and <0.5% of dairy and beef herds were antibody positive during 1998–2010 (23, 24). A nationwide voluntary BVD herd classification program was launched in 1994, and the disease was classified as combatted in 1995. At first, the eradication of BVD progressed rather slowly (23, 24). The initial low prevalence and insidious nature of the infection influenced the motivation to control BVD both locally and nationally (23). Finally, a compulsory control program was implemented in 2004, and intensive antibody testing from BTM samples was performed in 2004–2010 to identify the remaining infected dairy herds. In antibody-positive herds, control and eradication measures were successfully undertaken, such as the restriction of ruminant movements, reporting of infection to relevant stakeholders, enhanced biosecurity measures, individual sampling, and the removal of PI animals followed by resampling (23, 24).

The governmental compulsory control program to prove an official disease-free status is based on nationwide annual antibody surveys performed by BTM sampling and serum sampling in slaughterhouses. The BTM survey consists of both random and risk-based sampling of dairy herds with elevated levels of abortions. In addition, samples are tested from animals intended for artificial insemination (14). All aborted fetuses sent for autopsy and laboratory analysis are tested for BVD by PCR, and serum samples from aborted cows are examined for BVD antibodies. According to the instructions of Animal Health ETT, all imported animals must be tested for BVD. Testing of the recipient cattle of imported embryos is also recommended, but it is difficult to control. The total numbers of samples and cattle herds tested for BVD are presented in Tables 1, 2.

BVD is a disease to be combated, and suspicions and detected cases are dealt with by regional and local official veterinarians.

#### Bluetongue

Bluetongue has never been reported in Finland (17), and Finland was given an official disease-free status in 2021 (12). Sampling for BT antibodies is targeted at suckler cow herds and is combined with surveillance for IBR and BVD. Suckler cows are more likely than other cattle to be kept outside and are thus more exposed to the relevant vectors. Since animals are slaughtered throughout the year, sampling is also carried out throughout the year. BT is a disease to be combated, and suspicions and detected cases are dealt with by regional and local official veterinarians.

#### Aujeszky's Disease

Finland is officially free of AD, and the disease has never been reported in domestic animals in Finland (12, 18). The disease in cattle is to be reported.

#### Epizootic Hemorrhagic Disease

Epizootic hemorrhagic disease (EHD) has never been reported in Finland (18). In the case of suspected EHD infection in a cattle holding based on symptoms or other reasons, the herd owner must without delay inform a veterinarian.

#### Mycoplasma Mycoides

Finland is free of contagious bovine pleuropneumonia, and the last case occurred in 1920. In the case of suspected infection in a cattle holding based on symptoms or other reasons, the herd owner must without delay inform a veterinarian.

#### Bovine Genital Campylobacteriosis

Bovine genital campylobacteriosis has never been detected in Finland (7). All aborted fetuses sent for autopsy and laboratory analysis to FFA laboratories are examined for *C. fetus* spp. v*enerealis*. According to Council Directive 88/407/EEC, breeding bulls are also tested for *C. fetus* ssp. v*enerealis*. Bovine genital campylobacteriosis is a disease to be reported.

#### Leptospirosis

Leptospirosis has never been reported in cattle in Finland. Breeding bulls are tested for *L. hardjo* as well as for *Leptospira pomona, Leptospira grippotyphosa, Leptospira sejro, Leptospira canicola*, and *Leptospira icterohaemorrhagiae* (14). Leptospirosis in animals is a disease to be reported.

#### Trichomonosis

*Tritrichomonas fetus* was last detected in Finland in 1952 (22). All aborted fetuses sent for autopsy and laboratory analysis are examined for *T. fetus*. According to Council Directive 88/407/EEC, breeding bulls are also tested for *T. fetus*. There is no control program for trichomonosis in Finland. *Tritrichomonas fetus* is a disease to be reported.

### Control of Endemic and Sporadic Cattle Diseases in Finland

#### Salmonella

**Figure 4:**
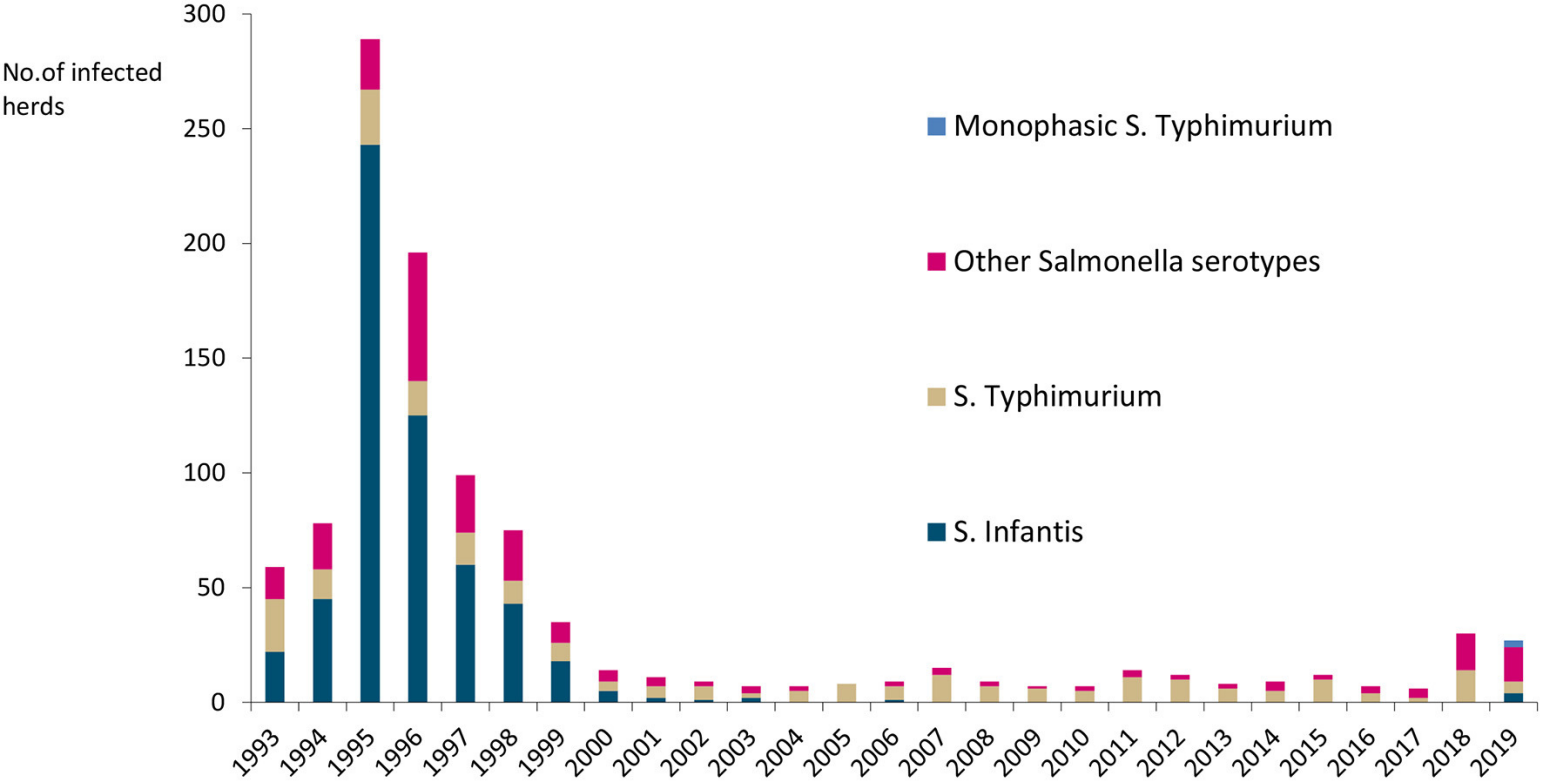
Number of *Salmonella*-infected cattle herds and serotypes in Finland in 1993–2019. No *Salmonella* Dublin was detected.

Salmonella occurs sporadically, with <0.5% of cattle herds infected annually, as illustrated in Figure 4. *Salmonella* Dublin has not been detected in cattle since a very few cases were reported in the 1980s (25). The most common serotypes have been *Salmonella* Typhimurium, monophasic *S*. Typhimurium, and *S*. Infantis. Finland has additional salmonella guarantees covering trade in fresh meat from bovine animals in the EU (26).

**Figure 5:**
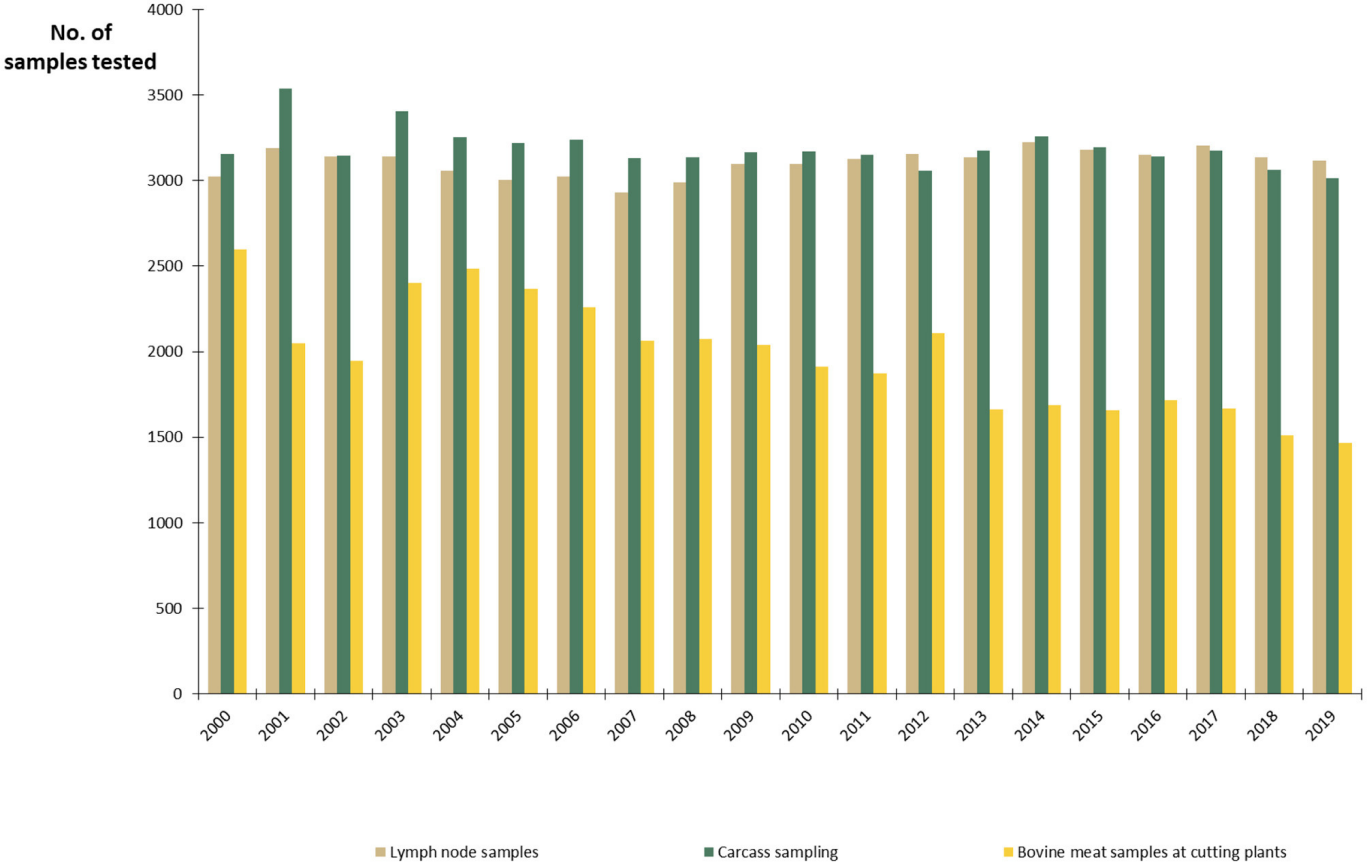
Number of samples from slaughterhouses and meat cutting plants annually tested for *Salmonella* in the control program in Finland in 2000–2019.

The Finnish National Salmonella Control Program, approved by the EC (27), has been in act since 1995. In cattle, the program covers live cattle and fresh meat. The aim is to minimize human exposure to *Salmonella* from production animals and foodstuffs by keeping the annual prevalence below 1%. Lymph node and carcass swab samples are taken at slaughterhouses and meat samples in meat cutting plants (Figure 5). The sampling is evenly distributed throughout the year. Herds sending cattle to semen collection or embryo production centers and herds delivering raw milk must be sampled for *Salmonella* (15). In all cattle herds, if there is any suspicion of *Salmonella* infection, for instance due to animal movements or clinical symptoms, sampling must be conducted. Furthermore, *Salmonella* control in the feed sector is an important part of successful *Salmonella* control. Manufactured, marketed, and imported feed materials and compound feeds are monitored by the FFA. Feed business operators must take own control samples from feeds and the processing environment, in addition to the official sampling.

In addition to the official control program, slaughterhouses, dairies, and food processing plants perform *Salmonella* testing as a part of their in-house control. Cattle herds belonging to the Naseva register are recommended to undertake annual fecal sample testing and testing of purchased cattle (Table 3). Herds participating in cattle shows should also be tested. During mandatory herd health visits, biosecurity measures and *Salmonella* sampling are discussed. Animal Health ETT maintains a positive list of the feed operators fulfilling additional criteria to ensure the safety of their products. Herds in the Naseva register must obtain feed from companies on the positive list.

Laboratories participating in the official control program must be approved by the FFA and accredited. Laboratories must send *Salmonella* isolates to the national reference laboratory (FFA) and inform food business operators, as well as regional and local official veterinarians, of the preliminary findings. The reference laboratory reports the confirmed results.

A herd that has tested positive or is suspected to be infected with salmonella is placed under official restrictions. These include the restriction of animal movements other than to slaughter, the delivery of milk only for pasteurization, and applying of biosecurity measures. An epidemiological investigation must be carried out by an official veterinarian to detect the infection source and to prepare a herd-specific eradication plan. The control measures depend on the extent of the infection, defined by the sampling of cattle, feed, and the environment. It is of major importance to ensure feed and feeding hygiene and thus to prevent the further spread of infection in the herd. Vaccination and the use of antimicrobials are not allowed. All major dairies and slaughterhouses have group insurance for their producers in case of *Salmonella*. The insurance covers most of the expenses of sanitation, eradication, and sampling costs during the eradication process on the farm.

To reverse the restrictions, the herd must be tested twice with negative results. Sampling is performed at intervals of 3–4 weeks on all animals, in pools of 20 at maximum. In addition, the environment (10–100 samples) must be sampled once. Salmonella is a disease to be combated, and official sampling and testing are financed by the government.

#### Mycoplasma Bovis

**Figure 6:**
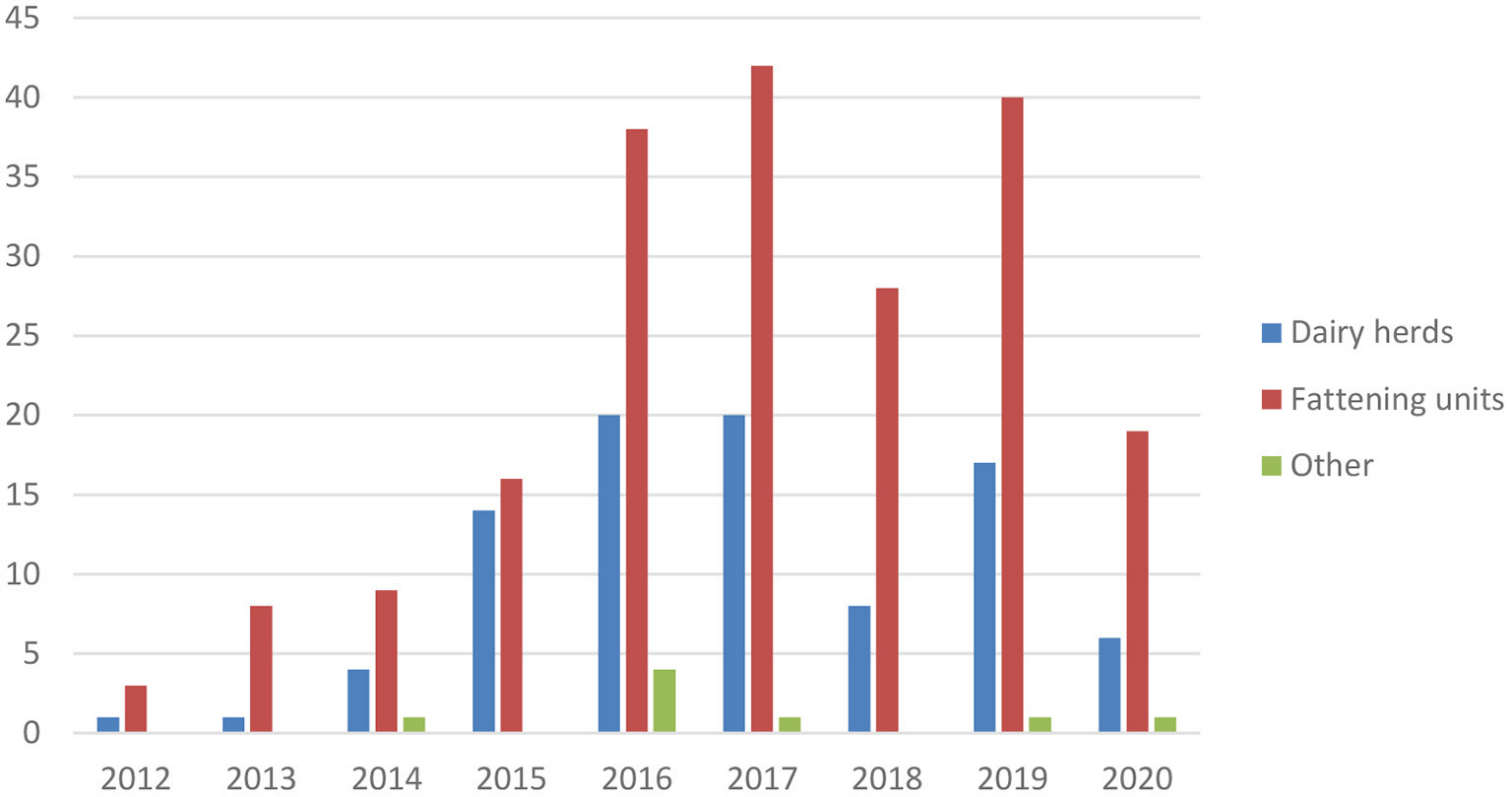
Number of new *M. bovis*-infected cattle herds in Finland in 2012–2020. Fattening units include calf rearing and finishing units.

*Mycoplasma bovis* has been endemic in Finland since the first detection in 2012 (7). The annual number of new cases is presented in Figure 6. The original infection source is unknown, but Finnish *M. bovis* strains resemble clones found in Denmark and Sweden (28). Contaminated bull semen was a source for some dairy herds (29). From dairy herds, the infection efficiently spreads *via* calves to calf-rearing or fattening units for meat production. Infections are mainly detected in mastitis QMS, samples in connection with clinical respiratory disease, or other clinical samples (30). In most dairy herds, the initial *M. bovis* case has been mastitis. The common testing of mastitis QMS by PCR helps in identifying *M. bovis*-infected dairy herds.

A national voluntary control program was established in 2013 and is administered by the Naseva register. The program aims to reduce the risk of introducing infection into dairy and suckler cow herds related to animal purchase, to improve animal welfare, and to reduce the use of antimicrobials in calf-rearing units. The key elements of the program are clinical monitoring and sampling of suspected cases, routine testing of mastitis agents (QMS), nasal swab sampling of calves, and control of animal trade. Slaughter results are also followed, as lung lesions are more common in infected herds (31). Farmers finance the costs of sampling, testing, and herd health visits.

**Figure 7:**
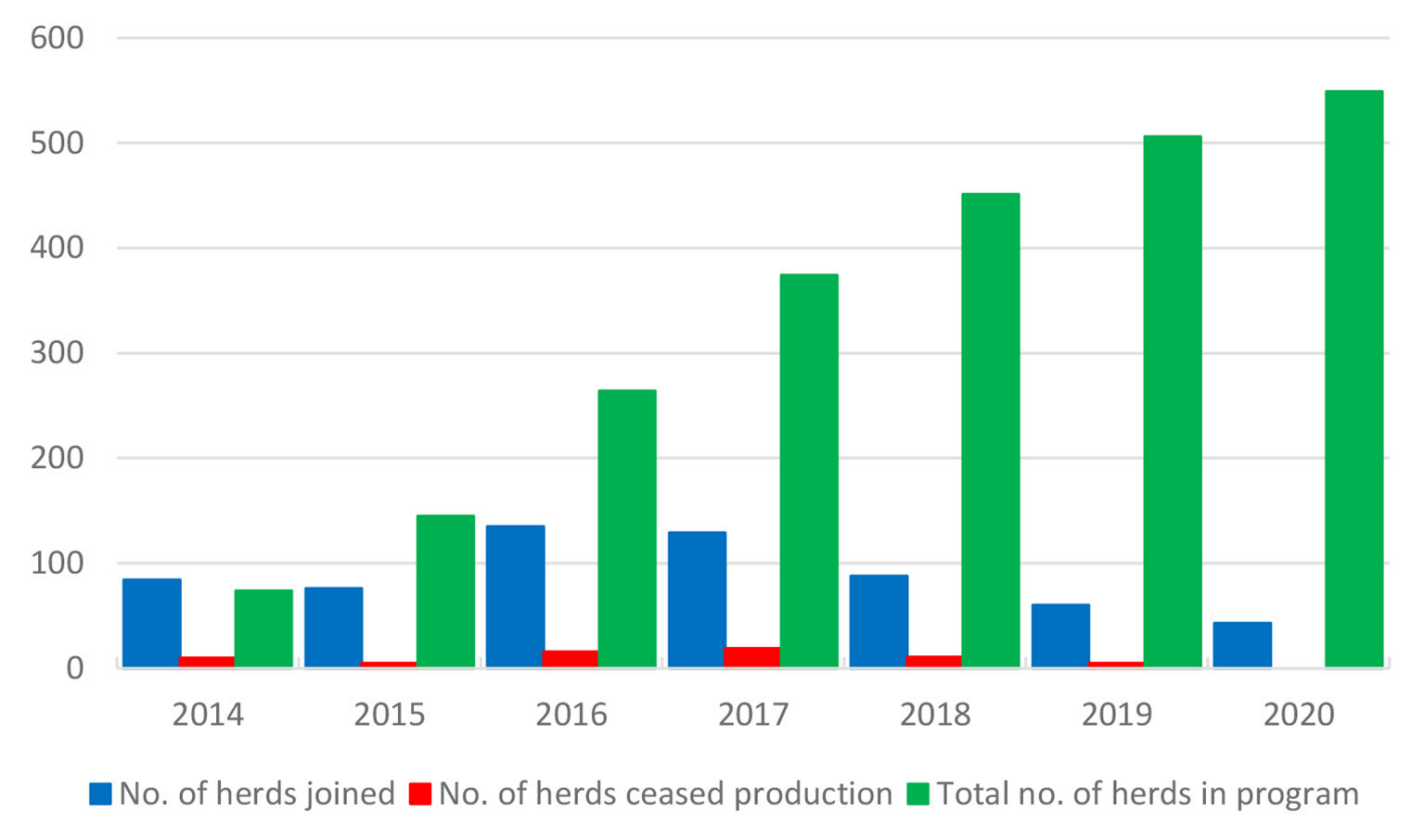
Number of herds that have annually joined and withdrawn from (ceased production) the program and total number of herds in the *M. bovis* control program in Finland.

The control program is described in Table 3 (32). The herds are categorized into levels A and B. There were 549 dairy and suckler cow herds in the program at the end of 2020 (Figure 7), corresponding to 8.5% of all dairy and suckler cow herds in the Naseva register, and a total of 377 dairy and 150 suckler cow herds had reached level A. A total of 66 herds have withdrawn from the program due to the cessation of production.

The herds at the Naseva national level may not be infected with *M. bovis* (Table 3). To support the control of *M. bovis* in infected dairy herds, the costs of testing during control measures are financed by the Naseva register. To control infection, it is advised to cull *M. bovis* mastitis cows and isolate the calves or prevent nose-to-nose contact with older animals (33). The calves are advised to be kept in a different air space, such as outdoor hutches, temporary pens, or in a different barn for at least 6 months. The infected herds reach the national level when they have consecutive negative results from regular QMS and BTM samples and on three sampling occasions at 4- to 8-month intervals for nasal swabs from calves (Table 3). In a total of 92 *M. bovis*-infected dairy herds, 46 have reached the national level, and control measures are ongoing in 46 dairy herds.

The *M. bovis* status of the herd is documented in the Naseva register and is available to authorized users, such as slaughterhouses, dairies, advisors, breeding organizations, and veterinarians. Health certificates are used when purchasing cattle or attending cattle shows. The purchase of cattle is only allowed from farms at the same or a higher level. *Mycoplasma bovis* A-level herds benefit from cattle trade of animals with a better health status. Therefore, small herds have joined the program to achieve level A and to obtain a better price for their cattle before ceasing production. Unfortunately, most herds (91.5% of Naseva register herds) have not found the program beneficial enough to join it. Most likely, these farms have not been infected with *M. bovis* and do not frequently trade animals or attend animal shows.

The program should be evaluated and improved with special emphasis on sampling and testing strategies. Proving freedom from infection with *M. bovis* is difficult. Nasal swabs are taken from several healthy calves, as the prevalence of *M. bovis* is thought to be low, and intermittent shedding of the agent is well-known. The control program does not specify the maximum number of samples that can be pooled, and there is no requirement to use an accredited method. *Mycoplasma bovis* antibodies persist in infected herds for a long time (30). The use of ELISA tests for *M. bovis* antibodies should be evaluated in presumably uninfected herds, as specific tests have become available (34). Similarly, ELISA tests could be used to follow how the infection level decreases in an infected herd during control measures. Overall, only accredited tests should be accepted in the control program.

*Mycoplasma bovis* is a disease to be reported and is not controlled by the government and veterinary authorities but by the industry.

#### Trichophyton Verrucosum

Ringworm is sporadic in the Finnish cattle population. It is detected in 20 to 30 new cattle herds annually (35), and a total of 2% of herds in the Naseva register are infected. The clinical signs, with typical skin lesions, define an infected animal. Clinical symptoms of ringworm are monitored during health visits to herds belonging to the Naseva register.

Since 2004, a national voluntary control program has been implemented (36). The program is run and financed by Animal Health ETT. The aim is to minimize the risk of infection at different stages of cattle production. Eradication of the disease is performed on infected dairy and suckler cow farms using vaccinations and hygienic measures according to a herd-specific plan approved by Animal Health ETT. All cattle in the herd are vaccinated twice, followed by subsequent vaccination of all calves born and animals purchased. The topical treatment of clinical cases is also advised. Farms are not allowed to sell animals to dairy or suckler cow herds for 3 years after the last clinical signs.

Half of the vaccine costs to the farmer are compensated. A disease-free status is achieved when the herd remains free of ringworm symptoms after finishing the vaccinations. Since 2004, a total 113 herds, comprising 96 dairy and 17 suckler cow herds, have participated in the program. Only three farms have failed to eradicate ringworm in the program.

The ringworm status of herds is documented in the Naseva register and is available to authorized users, such as slaughterhouses, dairies, advisors, breeding organizations, and veterinarians. Health certificates, with a statement of absence of ringworm symptoms, are used for animal trade and shows. Slaughterhouse animal brokers use this knowledge in the preselection of calf-rearing units for calves from infected farms to minimize the spread of infection in cattle-rearing units.

The disease is to be reported monthly.

#### Streptococcus Agalactiae

*Streptococcus agalactiae* is sporadic among Finnish dairy herds. A total of 114 (2%) herds in the Naseva register were classified as infected at the end of 2020. The control of *S. agalactiae* is based on extensive testing of QMS from cases of clinical and subclinical mastitis. Of ~170,000 mastitis QMS tested from herds in the Naseva register, only 0.7% harbored *S. agalactiae* in 2020.

Farms with *S. agalactiae*-positive herds are encouraged to eradicate the infection as soon as possible. The farmers pay the costs, but dairies may compensate the costs of testing during eradication. The key principle in eradication is the detection and culling or isolation of infected cows (37). To detect infected animals, all cows are tested for *S. agalactiae* by PCR using composite milk samples. In addition, all cows are tested post-partum for at least 1 year after the last *S. agalactiae* infection in the herd, and regular BTM sampling is conducted. The infected cows are either culled or isolated and subsequently treated with antibiotics. If infected cows are kept in the herd, they are milked separately and treated by intramammary infusion with benzylpenicillin or penethamate hydroiodide. The efficacy of treatment is evaluated by milk sample testing at 3 weeks post-treatment. Dry cow therapy is applied for all cows during eradication. Special attention is paid to the monitoring of subclinical and clinical mastitis, and milking hygiene in the herd. The environment as a reservoir for *S. agalactiae* should also be considered. The colostrum from infected cows is not given to newborn calves.

No data are available on the number of herds undergoing the eradication progress or on its success. The *S. agalactiae* status of the herd is documented in the Naseva register and is available to authorized users, such as dairies, advisors, breeding organizations, and veterinarians. Health certificates are used in animal trade and when attending shows.

The disease is not listed in the legislation.

#### Anthrax

Anthrax is rare in Finland. Since 1940, there have been 283 cases in 150 locations, the latest being in 2004 and 2008 (22, 38). There is no control program for anthrax, but it is a disease to be combated, and control measures are compulsory (39). In the case of suspected anthrax in a cattle holding based on symptoms or other reasons, the herd owner must without delay inform regional and local official veterinarians, and blood samples must be examined for *Bacillus anthracis* (39). Official restriction measures consist of the restriction of animal movements, isolation of diseased animals, clinical examinations, correct disposal of carcasses, decontamination of the site, and initiation of the treatment of other animals as appropriate.

#### Paratuberculosis

Paratuberculosis is rare in Finland and has never been reported in dairy herds. Thus, a control program for paratuberculosis has not been considered necessary. There have been some cases of paratuberculosis in beef suckler herds, the latest case being reported in 2000 (40). The symptoms of paratuberculosis are evaluated during annual health care visits to herds belonging to the Naseva register, and suspected cases must be sampled. A few herds have been annually tested for the presence of *Mycobacterium avium* subsp. *paratuberculosis* due to clinical suspicion, but with negative results. In 2020, in a nationwide study conducted among dairy and beef cattle, no positive herds were detected. Paratuberculosis is a disease to be reported and is not controlled by the government and veterinary authorities but by the industry.

#### Q-Fever

Q-fever is a rare disease in Finland, both in animals and in humans, and bovine abortions due to *Coxiella burnetii* have not been reported. In 2008, *C. burnetii* antibodies were detected in an animal tested for export. In subsequent testing, other seropositive cattle were found in the same herd, and *C. burnetii* was demonstrated by PCR in a milk sample. Nationwide BTM surveys and serum sampling in slaughterhouses conducted in 2009 and 2018 revealed only a few dairy and beef herds with antibodies (22). Q-fever is a disease to be reported, and there is no specific control program.

#### Neosporosis

Neosporosis occurs sporadically among cattle in Finland. Abortions caused by *Neospora caninum* occur in a few herds every year, and antibodies are detected in <10 cattle herds (7). Farms with positive herds are advised to control and eradicate the disease, but there is no control program for neosporosis. The disease is not listed among the disease categories in Finnish legislation.

#### Liver Fluke

Sporadic cases of liver fluke, *Fasciola hepatica*, occur in Finland. Meat inspection has reported lesions in <0.08% of cattle carcasses annually (41). In a nationwide survey of BTM and serum samples from slaughterhouses conducted in 2018, only a few dairy and beef herds had antibodies (22). Liver fluke is a disease to be reported, and there is no specific control program.

#### Staphylococcus Aureus

*Staphylococcus aureus* is endemic in dairy herds, and it is the second most common causative agent of mastitis in Finland (42). Roughly 20% of mastitis QMS harbor *S. aureus* (9, 42, 43). The control of *S. aureus* mastitis is the greatest challenge facing the Finnish dairy sector (33). Even though there is no specific control program, good milking practices and hygiene, routine PCR testing of mastitis QMS, and culling of carrier cows have reduced the proportion of penicillin-resistant *S. aureus* from 52% to 23% (2001–2012) (44, 45). According to the Naseva register, ß-lactame resistance has remained at the same level, being 24% in 2020. The disease is not listed in the legislation.

#### Bovine Coronavirus

Bovine Coronavirus is endemic in Finland (7). No specific control program for bovine coronavirus currently exists in Finland. Winter dysentery is to be reported monthly.

#### Bovine Respiratory Syncytial Virus

Bovine respiratory syncytial virus (BRSV) is endemic in Finland (7). No specific control program for BRSV currently exists in Finland. The disease is not listed in the legislation.

#### Bovine Digital Dermatitis

DD is endemic in Finland. Based on hoof trimming records, the prevalence of active lesions at the animal level was 2% in 2019 (46), and the herd-level prevalence of M2 lesions in freestall dairy herds was 12% in a recent research project (47). The diagnosis of DD is currently based on clinical signs. The lesions are detected while checking lame cows, during milking, or during hoof trimming. Good farm hygiene, the early detection and treatment of active lesions, and regular hoof bathing and hoof trimming are important control measures. DD is taken into account in health certificates used in cattle trade.

There is no eradication or control program for DD, and the disease is not listed in the legislation.

## Discussion and Conclusions

There are over 20 cattle diseases that are listed under category C, D, and E of the European Animal Health Law, and for which two or more regions in Europe have locally applied control programs (1). Here, we have described how these cattle diseases are controlled in Finland. Several cattle diseases have either been successfully eradicated from Finland, such as IBR, BVD, and EBL, or have never been detected in the country. Moreover, the control of *Salmonella* infections has been successful, and several other diseases occur only sporadically or at a low prevalence.

The key factors creating a good cattle disease situation include national disease control, nationwide screening of causative agents, the existence of national control programs, and limited and controlled import of live cattle. Active cooperation between authorities, the cattle industry, the industry-based association Animal Health ETT, and herd health experts, among others, enables efficient control and eradication, as well as the implementation of control programs. A characteristic of Finnish control programs, both compulsory and voluntary, is that they are national, not regional.

Overall, there are several control programs for cattle diseases in Finland compared to other EU countries. However, in contrast to other EU countries, there is no control program for paratuberculosis. This disease has only been detected in suckler cow herds, with the latest case in 2000. According to Finnish regulation, paratuberculosis is to be reported, but disease control is performed by the cattle industry and a control program has not been considered necessary. Similarly, in the case of BVD, eradication was rather slow, as the initial low prevalence and insidious nature of the infection influenced the motivation to control BVD on a voluntary basis. After implementing a compulsory control program, the disease was finally eradicated.

Even though the cattle disease situation is currently favorable in Finland, new agents may be introduced into country, which happened with *M. bovis* in 2012. Similarly, the prevalence of a rare disease may increase due to changes in cattle production, such as an increase in herd size, or climate change. Therefore, periodic active monitoring of non-regulated diseases is included in the national disease-monitoring program. Based on these monitoring studies, the need for control measures can be assessed in cooperation with national experts at the FFA and Animal Health ETT. Early detection of diseases by efficient passive surveillance, including a preference for autopsy samples submitted to the FFA, is an important part of the disease-monitoring program.

## Data Availability Statement

The original contributions presented in the study are included in the article/supplementary material, further inquiries can be directed to the corresponding author.

## Author Contributions

TA was responsible for drafting the manuscript. All the authors participated in collecting data, writing, and revising the manuscript and approved the submitted version.

## Conflict of Interest

The authors declare that the research was conducted in the absence of any commercial or financial relationships that could be construed as a potential conflict of interest.

## Publisher's Note

All claims expressed in this article are solely those of the authors and do not necessarily represent those of their affiliated organizations, or those of the publisher, the editors and the reviewers. Any product that may be evaluated in this article, or claim that may be made by its manufacturer, is not guaranteed or endorsed by the publisher.
