# Corrigendum: Overview of Control Programs for Cattle Diseases in Finland

**DOI:** 10.3389/fvets.2022.854052

**Published:** 2022-02-08

**Authors:** Tiina Autio, Erja Tuunainen, Hannele Nauholz, Hertta Pirkkalainen, Laura London, Sinikka Pelkonen

**Affiliations:** ^1^Finnish Food Authority, Veterinary Bacteriology and Pathology Unit, Kuopio, Finland; ^2^Animal Health ETT, Seinäjoki, Finland; ^3^Finnish Food Authority, Virology Unit, Helsinki, Finland

**Keywords:** cattle diseases, control program, SOUND control, Finland, bovine

In the original article, we used the phrase “non-regulated” for cattle diseases that are in fact listed in the New Animal Health Law that went into force in 2021. The corrected **Title**, **Short Running Title**, **Keywords**, **Abstract**, paragraph 4, **Introduction**, praragraph 3, **Categorization of cattle diseases in legislation**, paragraph 1, paragraph 2, the title of **Control programs for non-EU-regulated cattle diseases in Finland** section, **Discussion and conclusions**, paragraph 1, paragraph 3 and Figure 1 caption appear below.

A correction has been made to the **Title**:

“Overview of Control Programs for Cattle Diseases in Finland”

A correction has been made to the **Short Running Title**:

“Control Programs for Cattle Diseases in Finland”

A correction has been made to the **Keywords**, page 1:

cattle diseases, control program, SOUND control, Finland, bovine

Corrections have been made to the Abstract, paragraph 4, page 1:

CPs have been implemented for cattle diseases such as salmonella, *Mycoplasma bovis*, ringworm, and *Streptococcus agalactiae*. The CP for salmonellosis is compulsory and includes all Salmonella serotypes and all cattle types. It has achieved the goal of keeping the salmonella prevalence under 1% of cattle herds. CPs for *M. bovis*, ringworm, and *S. agalactiae* are on a voluntary basis and privately funded. The CP for *Mycoplasma* was designed in collaboration with national experts and has been implemented since 2013. The CP includes observation of clinical signs, nasal swab sampling from calves, and bulk tank milk and clinical mastitis samples for *M. bovis*. *M. bovis*-negative herds gradually achieve lower status levels for *M. bovis* infection. The general challenge facing voluntary CPs is getting farms to join the programs.

Corrections have been made to the Introduction, paragraph 3, page 2:

In this paper, we describe the current Finnish control measures and control programs for cattle diseases for which control programs have been implemented in two or more regions in the EU (1). The diseases were selected in the framework of the SOUND control project (COST Action Standardizing Output-based Surveillance to Control Non-Regulated Diseases in the EU, https://sound-control.eu). We also present the characteristics of disease surveillance and cattle production in Finland, which have enabled the good cattle disease situation.

Corrections have been made to the Categorization of cattle diseases in legislation, paragraph 1, page 2 and paragraph 2, page 3:

Cattle diseases fall into different control categories in Finnish legislation. All cattle diseases listed under category A, B, and C of the European Animal Health Law are controlled by the government, and reimbursements are paid to farmers if animals are culled. Of the diseases listed in categories C–E, the most important in the country are categorized as “to be combated” according to legislation, including IBR, EBL, BVD, BT, anthrax, and salmonella. These are controlled by the government, and for some of them, reimbursements are paid for the culled animals. If a disease to be combated is suspected on a cattle holding, the herd owner must inform a competent veterinary authority in accordance with the Animal Diseases Act 476/2021 (10). If a positive animal is detected, control measures are applied, including the restriction of animal movements and culling of cattle in the herd, depending on the disease.

Some diseases regarded as less serious in the country fall into the category “to be reported” by the veterinarian to the competent authorities. These are voluntarily controlled by the farmer and there is sometimes a voluntary control program or other control measures organized by the industry (such as for *Mycoplasma bovis*, paratuberculosis, and ringworm).

Corrections have been made to the title of **Control programs for non-EU-regulated cattle diseases in Finland** section, page 7:

“Control programs for cattle diseases in Finland”

Corrections have been made to the Discussion and conclusions, paragraph 1, paragraph 3, page 13:

There are over 20 cattle diseases that are listed under category C, D, and E of the European Animal Health Law, and for which two or more regions in Europe have locally applied control programs (1). Here, we have described how these cattle diseases are controlled in Finland. Several cattle diseases have either been successfully eradicated from Finland, such as IBR, BVD, and EBL, or have never been detected in the country. Moreover, the control of *Salmonella* infections has been successful, and several other diseases occur only sporadically or at a low prevalence.

Overall, there are several control programs for cattle diseases in Finland compared to other EU countries. However, in contrast to other EU countries, there is no control program for paratuberculosis. This disease has only been detected in suckler cow herds, with the latest case in 2000. According to Finnish regulation, paratuberculosis is to be reported, but disease control is performed by the cattle industry and a control program has not been considered necessary. Similarly, in the case of BVD, eradication was rather slow, as the initial low prevalence and insidious nature of the infection influenced the motivation to control BVD on a voluntary basis. After implementing a compulsory control program, the disease was finally eradicated.

Corrections have been made to the Figure 1 caption, page 3:

Figure 1. The latest cases of important cattle diseases and achievements in cattle disease control in Finland since 1950.

The authors apologize for these errors and state that they do not change the scientific conclusions of the article in any way. The original article has been updated.

## Publisher's Note

All claims expressed in this article are solely those of the authors and do not necessarily represent those of their affiliated organizations, or those of the publisher, the editors and the reviewers. Any product that may be evaluated in this article, or claim that may be made by its manufacturer, is not guaranteed or endorsed by the publisher.

